# Do We Need Another CT Scanner?—The Pilot Study of the Adoption of an Evolutionary Algorithm to Investment Decision Making in Healthcare

**DOI:** 10.3390/tomography9020063

**Published:** 2023-04-05

**Authors:** Katarzyna Kolasa, Grzegorz Kozinski, Maria Wisniewska, Aleksandra Pohadajlo, Agata Nosowicz, Piotr Kulas

**Affiliations:** 1 Division of Health Economics and Healthcare Management, Kozminski University, 03-301 Warsaw, Poland; 2 Ministry of Health, 00-952 Warsaw, Poland

**Keywords:** CT, machine learning, health economics, access to healthcare, health policy, retrospective database analysis

## Abstract

**Objectives**: The purpose of this study was to assess the feasibility of the adoption of a machine learning (ML) algorithm in support of the investment decisions regarding high cost medical devices based on available clinical and epidemiological evidence. **Methods**: Following a literature search, the set of epidemiological and clinical need predictors was established. Both the data from The Central Statistical Office and The National Health Fund were used. An evolutionary algorithm (EA) model was developed to obtain the prediction of the need for CT scanners across local counties in Poland (hypothetical scenario). The comparison between the historical allocation and the scenario developed by the EA model based on epidemiological and clinical need predictors was established. Only counties with available CT scanners were included in the study. **Results**: In total, over 4 million CT scan procedures performed across 130 counties in Poland between 2015 and 2019 were used to develop the EA model. There were 39 cases of agreement between historical data and hypothetical scenarios. In 58 cases, the EA model indicated the need for a lower number of CT scanners than the historical data. A greater number of CT procedures required compared with historical use was predicted for 22 counties. The remaining 11 cases were inconclusive. **Conclusions**: Machine learning techniques might be successfully applied to support the optimal allocation of limited healthcare resources. Firstly, they enable automatization of health policy making utilising historical, epidemiological, and clinical data. Secondly, they introduce flexibility and transparency thanks to the adoption of ML to investment decisions in the healthcare sector as well.

## 1. Introduction

To ensure the optimal allocation of scarce healthcare resources, any public budget spending should be justifiable from the clinical, epidemiological, and financial perspectives. On the one hand, the planning of investments in healthcare infrastructure must consider timely patient access, limiting waiting times whenever possible. On the other, an excessive number of medical devices may induce the risk of over utilisation. 

Across many jurisdictions, the conundrum of high demand vs. high cost exists in health policy planning and coordination mandated by law, particularly when public funds are involved. Thus far, there have been limited efforts with respect to the development of reimbursement schemes for high-cost medical equipment in a fashion similar to those already established for pharmaceuticals and high-risk medical devices. It is, therefore, prudent to ask whether there is an opportunity to introduce any new solutions to that problem in the era of growing data availability and AI-led methodological advancements. With this in mind, our study aimed to verify the feasibility of adapting a novel machine learning technique to the complex decision-making processes of health policy planning of high-cost medical devices. In particular, we sought to develop a data-driven support tool to optimise the allocation of computed tomography (CT) scans across geographical areas based on the assessment of clinical and epidemiological needs. 

The reason for the choice of CT scans was twofold. Firstly, it is a versatile imaging technique with a growing range of applications. Second, over the years, it has become one of the most important diagnostic methods, allowing for the accurate diagnosis of multiple indications across different branches of medicine. Its advantages and widespread use have made CT a very popular imaging technique. 

An evolutionary algorithm (EA) was chosen as the optimisation approach. EA is a flexible method derived from genetic algorithms (GAs) discovered in 1975 by Holland [1]. GAs were inspired by the process of biological evolution, including its many operators such as selection, crossover, and mutation. GAs and EAs are also used in medicine and healthcare research to solve different problems. However, to the best of our knowledge, evolutionary and GAs have not yet been used for the allocation of medical devices. 

## 2. Data Sources

Given the access to the relevant datasets, Polish settings were chosen for the pilot study. The project was conducted under the framework of a research agreement between Kozminski University and the Polish Ministry of Health (MoH). The statistics of CT procedures were derived from the anonymised National Health Fund (NHF) database containing records of all healthcare services utilised in the public healthcare system between 1 January 2015 and 31 December 2019 (historical data on file). The analysis was limited to CT scan utilisation, that is, CT scan referrals and procedures. The frequency of CT procedures was calculated for each record using the assigned ICD-10 code. The number of CT scanners and waiting times were sourced from the Polish MoH websites [2]. Since the data analysis was conducted at the county level, the database was structured with 380 Polish counties according to the geographical administrative setup of the country. However, the five largest Polish cities (with more than 0.5 million inhabitants) were further divided into municipalities (city districts) for our analysis. A total of 32 municipalities were included. Only counties with more than one CT scanner were selected for the study.

The demographic data with geographical distribution were obtained from available national statistics (The Central Statistical Office). 

## 3. Methods

The methodological approach involved two steps. 

The first step was to define the CT’s need predictors. Following the approach of other researchers, a set of epidemiological and clinical need predictors was established [3,4].

### 3.1. Epidemiological CT Need Predictors

Following the approach of Kung, P. T. et al. [4], a set of epidemiological need predictors were selected, including county population, female to male ratio, and populations over 65 and under 14 years old. Data on family income at the county level were not available; hence, this was omitted from the list of predictors [4]. The data were derived from The Central Statistical Office [5]. If available, the latest data from 2019 were used. Otherwise, the most recent statistics were obtained, i.e., for Wrocław (2018), Łódź (2017), and Poznań (2018). 

The analysis of each CT need predictor was conducted as follows: the counties were arranged in ascending order according to the length of the waiting time for the CT procedure and consequently divided into three groups (up to 25%, 25–75%, and above 75% of all counties). Basic statistics were separately calculated for all CT need predictors for each group. 

### 3.2. Clinical CT Need Predictors

The core set of the 50 most frequently used ICD-10 codes (indications) that accounted for at least 40% of all CT procedures was used to establish the clinical need predictors. The number 50 was arbitrarily selected based on the review of all CT procedures conducted in the study period to identify the most accurate break-even point for ICD codes ranked from the most to the least often medical condition mentioned (TOP 50).

Subsequently, the most recent international and national radiological guidelines were adopted to verify the appropriateness of CT utilisation for each indication of the core set of 50 ICD-10 codes. The guidelines of the Polish Society of Oncology, the Polish Neurological Society, and the Association of Polish Surgeons were regarded as the primary references. The recommendations of the European Society for Medical Oncology, the European Association for Cardiothoracic Surgery, the European Alliance of Associations for Rheumatology, the European Association of Urology, the European Federation of Neurological Societies, the Global Initiative for Asthma, and the American Stroke Association were used if a particular indication was not found in Polish patients [6,7,8,9,10,11,12,13,14,15,16,17,18,19,20,21,22,23,24,25,26,27,28,29,30,31,32,33].

By adapting the appropriateness rating scale developed by the American College of Radiology (ACR) for radiological procedures, each of the most common 50 ICD-10 codes was further broken down into four categories:

- Green—where CT use is ‘usually appropriate’;
- Yellow—where CT use ‘may be appropriate’;
- Red—where CT use is ‘usually not appropriate’.

The yellow category was assigned only when the ICD-10 code did not clearly indicate a specific medical condition, coding symptoms, or disorders not classified elsewhere. For each green indication, the number of patients was calculated at the county level to measure the clinical demand for CT scanners in the analysed areas. 

The second step was to develop an EA model of evolutionary data.

A two-step approach was used to verify whether the machine learning algorithm has the ability to recommend a different scenario for CT scan utilisation compared with the actual one (historical data).

First, the EA was designed to determine a hypothetical scenario of the distribution of CT scanners across counties in 2019. The fitness function was developed with epidemiological need predictors and the number of patients referred for the CT procedure per county for the study period. The following steps were performed.

1. The generation of 100 chromosomes was calculated. Each chromosome represented a different scenario for the distribution of CT scanners across the country and consisted of 130 genes representing the number of CT scanners per county.
2. The fitness function for each generation of chromosomes was calculated. The proposed custom fitness gave a score for each chromosome by assessing its probability of meeting the defined need using the set of epidemiological and clinical indicators. A lower score was assigned if a given chromosome met each need indicator to the lowest extent possible. The function was constructed based on the principle of a weighted sum of mean squared error (MSE) values between a normalised (from 0 to 1) series of particular data corresponding to the proposed distribution of CT scanners. The formula for the fitness function is given as
  $$f_{\mathrm{fitness}}\left(\mathrm{chromosome}\right)=w_{\mathrm{females}}p_{\mathrm{females}}+{\;w}_{\mathrm{old}}p_{\mathrm{old}}+{\;w}_{\mathrm{young}}p_{\mathrm{young}}+{\;w}_{\mathrm{population}}p_{\mathrm{population}}+{\;w}_{\mathrm{patients}}p_{\mathrm{patients}}$$
  where $w_{x}$ are weights for each x that is a CT need predictor, and $p^{\prime }{}_{x}$ values are defined as follows:
  $$p_{\mathrm{females}}=\mathrm{MSE}\left(v_{\mathrm{females}}^{\prime },{\;v}_{\mathrm{scanners}}^{\prime }\right)$$
  $$p_{\mathrm{old}}=\mathrm{MSE}\left(v_{\mathrm{old}}^{\prime },{\;v}_{\mathrm{scanners}}^{\prime }\right)$$
  $$p_{\mathrm{young}}=\mathrm{MSE}\left(v_{\mathrm{young}}^{\prime },v_{\mathrm{scanners}}^{\prime }\right)$$
  $$p_{\mathrm{population}}=\mathrm{MSE}\left(v_{\mathrm{population}}^{\prime },v_{\mathrm{scanners}}^{\prime }\right)$$
  $$p_{\mathrm{patients}}=\mathrm{MSE}\left(v_{\mathrm{patients}}^{\prime },v_{\mathrm{scanners}}^{\prime }\right)$$
  where $v_{\mathrm{females}}^{\prime }$, $v_{\mathrm{old}}^{\prime }$, $v_{\mathrm{young}}^{\prime }$, $v_{\mathrm{population}}^{\prime }$, $v_{\mathrm{patients}}^{\prime }$, and $v_{\mathrm{scanners}}^{\prime }$ are a series of female ratios, elderly people ratios, young people ratios, populations, and the number of patients with referrals for CT procedures per county during the study period, respectively, all scaled into a range from 0 to 1, according to the following method:
  $$v_{i}^{\prime }=\frac{v_{i}-\min\left(v\right)}{\max\left(v\right)-\min\left(v\right)}$$
  where $v_{i}^{\prime }$ is the scaled i-th element of a vector, $v_{i}$ is the unscaled i-th element of the vector, and v is the entire vector. Initially, the authors assumed setting different values of weights for each aspect based on domain knowledge and a series of tests; however, the algorithm exhibited very low sensitivity to different weights. Therefore, all weights were the same for all aspects and were equal to 0.1.
3. The chromosomes included in the subsequent generation were selected based on ‘roulette wheel selection’, which took the fitness score into account. This method assumed that the likelihood of being selected was proportional to the ratio of the fitness function score of a given chromosome to the sum of the scores of the entire generation. The lower the score, the greater the likelihood of being selected for the next generation. Because the purpose of optimisation is to lower the fitness function, inverses of the fitness function values were used.
4. After choosing chromosomes for a new generation, crossover between randomly chosen chromosomes occurred. This mechanism allowed for the exchange of information between chromosomes within the previous generation. Chromosomes were chosen for crossover with a probability of 30% (as suggested in [34]). Specific genes were exchanged between two random chromosomes with a probability of 10% per gene. This number was assumed to achieve approximately 10% of the entire chromosome informational content exchanged.
5. Mutation was used as the subsequent mechanism, with a probability of 0.5% [34] per gene. A random number from the range −5–10 was added to the number of CT scanner mutations in each gene. This range was adopted after the initial analysis of the number of scanners across the population of the counties. The higher limit (10) was set to a value higher than the lower limit (−5) to make it possible for some counties to increase their numbers faster. In cases where the added number of CT scanners would result in a negative number, a zero value was entered. This completed the set of tasks conducted for each generation.
6. The procedures outlined in steps 1–5 were repeated until satisfactory results were obtained, that is, when the fitness function could not be decreased further. Previous tests showed that for particular settings, the number of generations was set to 1000.

Second, a feasibility study for the EA model was constructed to validate it against the available data on waiting time for the CT procedure. Counties were categorised into one of the following groups according to the following:

1. The groups of counties were ranked in ascending order from the shortest to the longest waiting times for a CT scan (waiting time = the number of waiting days).
2. Waiting times were divided into three groups:
  - Q1—waiting time ranging from 0 to 25% of the longest waiting time across included counties;
  - Q2—waiting time ranging from 25% to 75% of the longest waiting time across included counties;
  - Q3—waiting time ranging from 75% to 100% of the longest waiting time.
3. The counties were grouped into one of the following categories based on the difference between the historical number of CT scanners in a given county and the result from the EA model and additionally compared with the average waiting times to obtain CT scans for each county.
  - EA predicted a greater number of CT scans compared with historical data, and the average waiting time was in Q3—underinvestment of CT.
  - EA predicted a greater number of CT scans compared with historical data and the average waiting time was in Q2—potential for further development of equipment infrastructure.
  - EA predicted a lower number of CT scans compared with historical data, and the average waiting time was in Q3—potential for efficiency gains.
  - EA predicted a lower number of CT scans compared with historical data, and the average waiting time was in Q2—overuse of CT.
  - EA predicted a greater number of CT scans compared with historical data, but waiting times were at the average level in Q2—underuse of CT scanners.
  - EA predicted a lower number of CT scans compared with historical data, but waiting times were at the average level in Q2—overuse of CT scanners.
  - EA predicted a similar number of CT scans as that currently available in a given county.

## 4. Results

More than 4.7 million CT scans were used with at least one of the TOP 50 ICD codes, which is 41% of the total performed in the study period (January 2015–December 2019). The breakdown of CT procedures with respect to the green, yellow, and red categories is presented in Supplementary Table S1. 

Only counties with at least one CT scanner were considered for the study. Out of 412 countries, only 130 were included. The descriptive characteristics with historical data on CT utilisation are presented in Table 1.

The pool of patients referred for the CT procedure was four times higher in the group of counties with the longest (Q3) vs. shortest (Q1) waiting time (Table 1). The average number of CT scanners per county was 3.1 and 8.7 for the group with the shortest (Q1) and longest (Q3) waiting time, respectively. The average size of the county population was 109,508 and 158,398 in the first and last groups, respectively (Table 1). In addition, the group of counties with the longest (Q3) waiting times had about two times more CT scans performed in comparison with the counties with the shortest (Q1) waiting times; on average, these were 15,192 and 37,023, respectively (Table 1).

The result of the EA model was a chromosome that achieved the lowest possible fitness score across all generations. Table 2 presents the results for each county. The number of iterations was stopped at 1000 generations, as further runs showed that the fitness function scores did not significantly decrease. 

The descriptive characteristics of each of the seven groups are presented in Table 3. It should be noted that 11 counties were not assigned to any of the predefined groups (some of the counties were assigned to other categories, but due to the substantial insignificance of those categories, they were excluded from the analysis). 

In the feasibility study, the results for 119 counties were included. Among these, there were 39 cases with EA predictions comparable to the historical data (Group G). In 58 counties (Groups C, D, and F), EA indicated a lower number of CT scans than the historical data. A greater need for CT examination compared with historical use was predicted in 22 counties. The results for all CT need predictors for each group are presented in Table 3.

## 5. Sensitivity Analysis

Sensitivity analysis with the chosen chromosome was conducted. Because randomness is strictly involved in evolutionary optimisation, the results were also affected.

An analysis of the sensitivity of weights also showed a low response of the results to changes in weights, which were initially assumed to vary to test different domain knowledge-based scenarios. Table 4 presents the results for different weight values and shows that the resulting values did not significantly differ from the previous ones, where all weights were equal to 0.1. The only exception is the scenario where the highest weight was applied to the population of counties. For this scenario, the results are noticeably different from other cases, which suggests that this factor should have the highest influence on the results.

## 6. Discussion

The purpose of this study was to assess the feasibility of adapting machine learning to decision-making regarding the allocation of high-cost diagnostic medical devices. The investment process with respect to computed tomography (CT) was chosen for that study. Access to CT scanners is considered vital for ensuring prompt and accurate diagnostics, allowing for timely treatment. Our analysis provided evidence that EAs may be a useful approach in that respect. Three interesting findings are worth highlighting.

First, the EA model was responsive to epidemiological need predictors for the allocation of CT scanners. When a similar number of CT scanners were predicted by the model as historical data (Categories A, E, and G), there was consistency with respect to key measures such as the mean, median, minimum, and maximum for each predictive variable. 

Second, the EA model was validated against the waiting time for CT scans. It must be noted that waiting times were not used during the ML algorithm development. It was found that there is a tendency for EA to predict a greater number of CT scans in counties with longer waiting times. With a median of 63 days of waiting, EA indicated a median of five CT scanners per county compared with two CT scanners per county for a category with a median of six waiting days. 

Third, our study proved that the adoption of the EA approach can aid allocative decisions by considering more determinants of the need for CT investment than waiting time alone. Interesting findings emerge from the comparison of Category G, which comprises counties with a historical number of CT scans similar to EA predictions against other groups. For example, Category C has epidemiological characteristics similar to those of Category G. Hence, EA predicted a similar number of CT scanners in both groups. However, the historical data clearly indicate that the number of CT scanners is greater than that predicted as optimal by EA in Category C. Thus, adding epidemiological predictors into decision-making can validate the rationale behind waiting times and even indicate potential efficiency gains in the case of underutilisation of available CT scanners. Interesting patterns with respect to both epidemiological and clinical need predictors were also observed for Categories G and E. In the latter, however, the mean values of the predictions were slightly higher, which justifies the greater number of CT scanners predicted by the EA model. The historical number of available CT scanners remained similar for both categories. In that particular example, it is noteworthy that the use of an algorithmic approach based on epidemiological predictions could support the decision to increase the allocation of CT scanners for counties in Category E, even though the current waiting times alone would not justify such a decision.

This study was not the first attempt to adopt EAs in the field of healthcare. However, there are examples of research efforts focusing on other types of problems. Gao et al. used evolutionary algorithms to diagnose bradykinesia in finger-tapping tests [35]. EAs were used to develop a classifier algorithm that correctly classified the test results of patients with early stage Parkinson’s disease. In 2019, Haddadene et al. successfully used a non-dominated sorting genetic algorithm for the first time to solve the vehicle routing problem for home healthcare for scheduling caregivers’ visits to their patients under specific conditions such as time, place, and patient preference [36]. The problem had the form of multi-objective combinatorial optimisation, with the first objective being minimising travel cost and the second objective being maximising patient satisfaction. He used evolutionary algorithms for the optimisation of resource allocation in the steel forging process [37]. The steel forging process can be described as a hybrid flow shop scheduling problem, and in that research, bi-objective evolutionary algorithms were successfully used along with real industrial data. Nanbin et al., in turn, used a hybrid system of greedy algorithms and GAs for resource allocation in the energy sector [38]. 

The problem of decision-making regarding the allocation of high-cost diagnostic medical devices has been addressed with different approaches. In 1978, Hosios et al. proposed a linear programming model that could be used by decision-makers for the allocation of radiographic equipment [39]. The production model of a diagnostic radiology department was created based on available data that included aspects such as medical and non-medical manpower (e.g., technicians or materials). Santibanez et al. used an integer programming model to solve the combinatorial optimisation problem of allocating radiation therapy centres based on geographic access in British Columbia, Canada [40]. Similarly, Czerwiński et al. used mixed-integer linear programming to allocate linear accelerators in Poland [41]. Tal et al. conducted questionnaire-based research among stakeholders of various medical-related backgrounds on aspects related to the allocation of expensive medical devices (EMD) [42]. The study showed that different criteria used for EMD allocation have different importance and, therefore, should be weighted differently in the decision-making process. A similar methodology was applied to the analysis of large medical equipment (LME) allocation in the Chinese province of Xuzhou in a study conducted by Miao et al. [43]. Research based on questionnaire answers received from local hospitals equipped with LME showed that the allocation of equipment was unfair.

Despite the uniqueness of our study, it is not free from limitations, at least five of which must be highlighted. First, only localities with more than one CT scanner were considered. The majority of counties (68.4%) had at most one CT scanner (97 localities had no CT scanners and 185 localities had only one CT scanner) and, thus, were excluded from the analysis. This approach was chosen to reduce the bias related to population distribution: such localities will usually have a relatively tiny population, but at the same time decrease heterogeneity (as larger differences in the selected demographic characteristics are expected between more urbanised and rural areas). Another important consideration is the practical difficulty of disinvestment if there is only one CT scanner in a particular county, as matters related to the distance for the patient and local policies play an important role. 

Second, only 27 indications (identified by their ICD-10 codes) of the TOP 50 were considered in this study. The final analysis included indications that appeared in only 18% of all CT referrals. Exclusion of ICD-10 indications in which a CT scan did not constitute a standard of care was a key part of the analysis, which aimed at identifying clinical needs and not preserving current clinical practice. At the same time, it should be recognised that the database used in the analysis includes only one ICD-10 code for each contact (called the main ICD-10 code), where only a symptom of a disease constitutes the basis for referral (as the root cause of the symptom might be unknown at the time). 

Third, 37 districts of the five largest cities (municipalities) were considered. Municipalities constitute 28% of all selected localities and have larger populations and a higher average number of CT scanners. Thus, an alternative approach to counties should be considered to ensure that the results better reflect patient mobility. The inclusion of city districts on par with counties leads to a situation where, in the case of two cities (i.e., Warszawa and Wroclaw), the algorithm suggests a larger number of CT scanners in some districts and an a smaller number in others. This effect is exacerbated when the concentration of care in large cities (e.g., major trauma centres and tertiary care centres) is taken into account. In the case of highly specialised healthcare providers, waiting times are sometimes significantly longer than in other nearby places (due to the provision of highly specialised care for the whole region). In this study, highly specialised care providers were not separately analysed. 

Fourth, in this study, no consideration was given to differences in morbidity and mortality across regions. As some studies have shown that morbidity-based models may capture more adequate health needs than utilisation-based models, this aspect requires further research [44,45]. 

Finally, three demographic criteria reflecting the size of the population were considered. Although the choice of demographic indicators was influenced by the literature review, the possible impact of choosing three indicators closely related to the population (including the populations over 65 and under 14 years of age) is unknown and could be an avenue for further investigation.

## 7. Conclusions

The presented methodology shows how a variety of attributes may be included in the decision-making process in the healthcare sector. The ML algorithm offers an opportunity to simultaneously categorise clinical and epidemiological need predictors and even compare results with historical data. Our study indicates that it is possible to adopt ML for reimbursement decision-making in the healthcare sector. The use of a single broad fitness function allows different aspects to be considered and translated into one numerical value based on selected domain knowledge. The function takes as input locations to be supplied with a particular type of equipment and returns a single numerical value of how well a given combination suits the needs, which can be used to create a score for a particular combination of possible locations. For example, distance might be one aspect; therefore, geographical data along with health needs predictors should be used to define the optimal number of medical devices given the combination of attributes. Historical data from previous use of CT scanners can be used to validate the fitness function itself. The use of ML algorithms not only introduces an objective evidence-based approach to the reimbursement process, but also allows for flexibility and adaptability to the given decision-making problem. Our study demonstrates how such an approach can be introduced. Medical records coupled with clinical guidelines will help identify which criterion has the greatest influence on the use of a particular medical device. The fitness function will be later used with the basic form of the EA using crossover and mutation as operators. After a fixed number of iterations (generations), the algorithm finds the best approximated solution. It is therefore an evidence-based approach to decision-making encompassing a broad spectrum of attributes.

One advantage of the proposed approach towards the allocation of new investment in the decision-making process is the convenience of the easy interpretability of the final results. Our model produces a quantifiable result, such as a given number of CT scanners that should meet the demand driven by unmet medical needs in each of the selected geographical areas. Consequently, such an approach has the potential to be used as a supporting tool in many different allocation problems utilising basic statistics beyond the investigation of the CT scanners used in this particular study.

In summary, the introduction of ML algorithms in the decision-making process may provide many benefits to the healthcare system in general.

First, it may provide external validation of already adopted investment decisions as well as indicate the geographical regions with under supply or over supply of CT scanners. The latter may provide new opportunities to identify budget savings without reducing the level of healthcare in a particular area.

Second, it is a flexible method that allows us to add new variables in case there is a significant change in the situation in the country requiring adjustments to the model. It is worth mentioning that it is crucial to conduct continuous verification of the results to quantify the model’s accuracy. Additional information obtained from such an analysis would enable us to improve the model in the future.

## Figures and Tables

**Table 1:** General characteristics of the Polish utilization of CT scanners for 130 counties included in the study categorized into three groups by the waiting time for CT procedure (2019).

Waiting Time (Quartile, Range)	Number of Counties (N)		Sum	Mean (SD)	Min	Max
**Short** (<Q1, 1–33)	33	Population of studied counties	3,613,753	109,507.7 (54,156)	35,193	297,554
		*% of female*		52.0 (1.1)	50.0	54.746
		*% of male*		48.0 (1.1.)	45.3	50.0
		*Above 65 years old*	691,161	20,944.3 (10,463.4)	5655	52,967
		*Below 14 years old*	526,187	15,945.1 (8638.8)	4648	45,189
		*Diagnosed with CT*	177,941	5392.2 (5474.1)	1252	25,340
**Medium** (Q1–Q2, 34–98)	65	Population of studied counties	8,060,044	124,000.7	24,513	348,190
		*% of female*		52.7 (1.2)	49.9	55.0
		*% of male*		47.3 (1.2)	45.0	50.1
		*Above 65 years old*	1,630,879	25,090.4 (15,423.1)	3992	75,715
		*Below 14 years old*	1,223,938	18,829.8 (9952.3)	4011	50,032
		*Diagnosed with CT*	552,536	8500.6 (9276.6)	171	43,420
**Long** (Q2–Q3, 99–130)	32	Population of studied counties	5,068,719	158,397.5	46,291	470,907
		*% of female*		52.4 (1.0)	50.4	55.0
		*% of male*		47.6 (1.0)	46.2	49.6
		*Above 65 years old*	1,010,497	31,578.0 (19,499.2)	9676	96,394
		*Below 14 years old*	743,630	23,238.4 (13,175.7)	6285	72,801
		*Diagnosed with CT*	389,340	12,166.9 (9214.3)	2022	37,407
**Short** (<Q1, 1–33)	33					
		Number of CTs	101	3.1 (2.0)	2	13
		*Number of CT procedures*	501,332	15,191.9 (21,253.3)	2011	97,837
		*Number of CT procedures per patient*	80.2	2.4 (0.7)	1.5	4.11
		*Number of patients waiting (urgent + stable cases)*	11,293	342.2	32	1169
**Medium** (Q1–Q2, 34–98)	65					
		Number of CTs	306	4.7 (3.9)	2	23
		*Number of CT procedures*	1,754,639	26,994.4 (37,161.9)	269	208,486
		*Number of CT procedures per patient*	176.4	2.7 (0.7)	1.5	4.8
		*Number of patients waiting (urgent + stable cases)*	102,686	1579.8 (1411.6)	100.0	4269
**Long** (Q2–Q3, 99–130)	32					
		Number of CTs	277	8.7 (9.1)	2	28
		*Number of CT procedures*	1,184,735	37,023.0 (32,218.1)	4049	129.82
		*Number of CT procedures per patient*	88.9	2.8 (0.6)	1.7	3.9
		*Number of patients waiting (urgent + stable cases)*	39,955	1248.6 (1036.9)	41	4914

**Table 2:** Number of CT scanners determined by the EA model (best chromosome) per each county (2019).

County’s Code	County’s Name	Number of CT Scanners Obtained from Algorithm	Current Number of CT Scanners
0202	dzierzoniowski	3	3
0206	jeleniogórski	1	2
0208	kłodzki	1	2
0211	lubiński	3	3
0219	świdnicki	2	2
0225	zgorzelecki	3	2
0261	City-Jelenia Góra	2	3
0262	City-Legnica	4	2
0264049	City-Wroclaw-Psie Pole District	4	ND
0264069	City-Wroclaw-Srodmiescie District	1	ND
0264059	City-Wroclaw-Old Town District	6	ND
0264039	City-Wroclaw-Krzyki District	4	ND
0264029	City-Wroclaw-Fabryczna District	4	ND
0264	City-Wroclaw	19	18
0461	City-Bydgoszcz	6	18
0462	City-Grudziądz	1	3
0463	City-Toruń	4	6
0464	City-Włocławek	3	3
0614	puławski	3	3
0661	City-Biała Podlaska	3	2
0662	City-Chełm	2	2
0663	City-Lublin	6	21
0664	City-Zamość	2	4
0804	nowosolski	2	2
0807	sulęciński	3	2
0808	świebodziński	2	3
0811	żarski	2	2
0861	City-Gorzów Wielkopolski	4	3
0862	City-Zielona Góra	4	3
1003	laski	3	2
1008	pabianicki	5	2
1016	tomaszowski	3	3
1017	wieluński	1	2
1019	zduńskowolski	2	2
1020	zgierski	3	5
1021	brzeziński	1	1
1061059	City-Lodz-Śródmieście District	2	8
1061069	City-Lodz-Widzew District	4	15
1061029	City-Lodz-Baluty District	6	10
1061039	City-Lodz-Gorna District	6	11
1061049	City-Lodz-Polesie District	5	9
1205	gorlicki	1	2
1207	limanowski	2	2
1211	nowotarski	2	4
1217	tatrzański	4	2
1261029	City-Krakow-Krowodrza District	6	8
1261039	City-Krakow-Nowa Huta District	6	6
1261049	City-Krakow-Podgorze District	7	4
1261059	City-Krakow-Srodmiescie District	4	14
1262	City-Nowy Sącz	2	2
1263	City-Tarnów	2	4
1405	grodziski	4	2
1407	kozienicki	0	2
1417	otwocki	2	5
1418	piaseczyński	5	4
1428	sochaczewski	3	2
1433	węgrowski	3	2
1462	City-Płock	1	3
1463	City-Radom	3	8
1464	City-Siedlce	1	3
1465078	City-Warsaw-Praga Południe District	3	11
1465168	City-Warsaw-Wilanów District	4	2
1465058	City-Warsaw-Mokotow District	5	15
1465158	City-Warsaw-Wesoła District	2	0
1465028	City-Warsaw-Bemowo District	4	0
1465198	City-Warsaw-Żoliborz District	3	5
1465098	City-Warsaw-Rembertów District	2	0
1465068	City-Warsaw-Ochota District	4	13
1465048	City-Warsaw-Bielany District	4	5
1465038	City-Warsaw-Białołeka District	2	0
1465128	City-Warsaw-Ursus District	2	1
1465088	City-Warsaw-Praga Północ District	2	5
1465138	City-Warsaw-Ursynów District	4	11
1465148	City-Warsaw-Wawer District	5	4
1465108	City-Warsaw-Śródmieście District	6	11
1465188	City-Warsaw-Wola District	3	4
1465178	City-Warsaw-Włochy District	3	1
1465118	City-Warsaw-Targówek District	5	2
1604	kluczborski	0	2
1802	brzozowski	2	4
1811	mielecki	3	2
1816	rzeszowski	2	2
1818	stalowowolski	2	2
1861	City-Krosno	2	2
1862	City-Przemyśl	3	2
1863	City-Rzeszów	3	12
2061	City-Białystok	7	11
2063	City-Suwałki	0	2
2202	chojnicki	1	2
2206	kościerski	2	2
2207	kwidzyński	2	2
2215	wejherowski	2	5
2261	City-Gdańsk	8	11
2262	City-Gdynia	6	3
2263	City-Słupsk	2	3
2401	będziński	4	2
2403	cieszyński	2	6
2405	gliwicki	2	3
2411	raciborski	4	2
2413	tarnogórski	5	2
2416	zawierciański	4	2
2461	City-Bielsko-Biała	3	6
2462	City-Bytom	5	6
2463	City-Chorzów	3	2
2464	City-Częstochowa	4	6
2465	City-Dąbrowa Górnicza	4	3
2466	City-Gliwice	4	5
2469	City-Katowice	6	18
2471	City-Piekary Śląskie	1	2
2473	City-Rybnik	2	2
2475	City-Sosnowiec	5	5
2477	City-Tychy	2	2
2478	City-Zabrze	5	4
2607	ostrowiecki	2	2
2610	skarżyski	2	2
2661	City-Kielce	3	9
2861	City-Elbląg	3	3
2862	City-Olsztyn	6	10
3017	ostrowski	3	2
3019	pilski	2	3
3020	pleszewski	2	2
3061	City-Kalisz	3	2
3062	City-Konin	3	3
3063	City-Leszno	2	3
3064049	City-Poznan-Nowe Miasto District	2	9
3064059	City-Poznan-Stare Miasto District	3	11
3064039	City-Poznan-Jezyce District	3	11
3064029	City-Poznan-Grunwald	1	6
3064069	City-Poznan-Wilda District	2	2
3261	City-Szczecin	4	4
3262	City-Świnoujście	10	11

Note: ND means no data.

**Table 3:** Descriptive characteristic of the studied Polish counties grouped into one of the seven categories based on the difference between the historical number of CT scanners in a given county and the results of the EA model and compared with historical average CT waiting times (2019).

	Female Ratio (%)	Under 14 Years Old (%)	Population over 65 (%)	County Population	CT Procedures	CT Scanners- EA Model	CT Scanners—Historical Data	Waiting Time
Group A (underinvestment of CT) (n = 9 counties)								
Mean	0.53	0.15	0.21	145,609	7596	4.89	3.67	69
Median	0.53	0.14	0.21	124,992	6513	5.00	3.00	64
Min.	0.52	0.11	0.17	78,244	1505	4.00	2.00	48
Max.	0.55	0.18	0.25	246,348	15,349	6.00	5.00	122
Group B (potential for further development of the equipment infrastructure) (n = 3 counties)								
Mean	0.51	0.15	0.19	91,641	3681	3.50	2.50	6.96
Median	0.51	0.15	0.19	91,641	3681	3.50	2.50	6.96
Min.	0.50	0.14	0.16	35,193	2237	3.00	2.00	5.00
Max.	0.52	0.16	0.21	148,089	5124	4.00	3.00	8.92
Group C (potential for efficiency gains) (n = 35 counties)								
Mean	0.53	0.16	0.19	156,436	11,660	3.56	9.44	61.56
Median	0.53	0.15	0.20	131,296	8541	3.00	5.00	53.45
Min.	0.50	0.12	0.09	24,513	171	0.00	2.00	47.89
Max.	0.55	0.26	0.24	483,789	38,896	10.00	28.00	129.12
Group D (overproduction of CT) (n = 5 counties)								
Mean	0.51	0.15	0.18	98,247	3083	1.40	2.80	6.24
Median	0.51	0.15	0.18	76,256	2859	2.00	3.00	6.00
Min.	0.51	0.13	0.17	55,790	1252	0.00	2.00	3.92
Max.	0.52	0.16	0.18	178,191	6569	2.00	4.00	9.14
Group E (underconsumption of CT scans) (n = 10 counties)								
Mean	0.53	0.15	0.19	146,319	6954	4.50	3.00	18.38
Median	0.52	0.15	0.19	129,905	4278	4.00	3.00	12.74
Min.	0.51	0.10	0.15	47,028	1380	4.00	2.00	11.93
Max.	0.55	0.19	0.25	308,070	24,076	6.00	4.00	40.23
Group F (overconsumption of CT scans) (n = 18 counties)								
Mean	0.52	0.15	0.19	222,157	16,481	3.00	8.44	29.86
Median	0.52	0.15	0.19	182,758	9106	2.50	6.00	28.84
Min.	0.51	0.12	0.14	64,760	2661	0.00	2.00	12.74
Max.	0.54	0.20	0.27	495,350	43,420	7.00	23.00	47.03
Group G (n = 39 counties)								
Mean	0.52	0.15	0.19	137,813	6190	2.79	2.77	25.64
Median	0.52	0.15	0.19	136,112	4931	3.00	2.00	26.11
Min.	0.50	0.12	0.13	30,833	1324	1.00	2.00	10.00
Max.	0.55	0.19	0.29	300,590	27,348	6.00	6.00	46.95
Unclassified (n = 11 counties)								
Mean	0.51	0.15	0.18	136,670	4728	2.15	2.15	43.22
Median	0.51	0.15	0.18	139,946	3790	2.00	2.00	48.42
Min.	0.50	0.13	0.15	63,014	1483	2.00	2.00	4.94
Max.	0.52	0.17	0.21	253,142	13,128	3.00	3.00	114.34

**Table 4:** Results of the weight sensitivity analysis—number of localities distributed to group A–G in runs of the model based on changed weights.

Need Predictor Weights					Number of Localities in Particular Groups						
Population size	Female ratio	Aged >64	Aged <14	CT patients	Group A	Group B	Group C	Group D	Group E	Group F	Group G
0.5	0.1	0.1	0.1	0.1	9	4	36	4	11	16	40
1	0.1	0.1	0.1	0.1	15	3	31	4	10	18	39
0.1	0.5	0.5	0.5	0.1	14	6	30	3	9	19	39
0.1	1	1	1	0.1	15	5	30	6	11	13	43

## Data Availability

Restrictions apply to the availability of these data. Data was obtained from Ministry of Health, Poland and are available at request from the corresponding author with the permission of Ministry of Health, Poland.

## Supplementary Materials

The following supporting information can be downloaded at: https://www.mdpi.com/article/10.3390/tomography9020063/s1, Table S1: TOP 50—The list ICD code qualified as Green (“usually appropriate”), Yellow (“may be appropriate”), Red (“usually not appropriate”).
