# Supplementary material for: Do We Need Another CT Scanner?—The Pilot Study of the Adoption of an Evolutionary Algorithm to Investment Decision Making in Healthcare
Source: Tomography. 2023 Apr 5;9(2):776–89. doi: 10.3390/tomography9020063 (PMC10141352; doi:10.3390/tomography9020063)
Supplement: Supplementary file 1 [file tomography-09-00063-s001.zip › tomography-2272477-supplementary.pdf]

Supplementary Table S1. TOP 50 - The list ICD code qualified as Green (“usually appropriate”), Yellow (“may be appropriate”), Red (“usually not appropriate”).

Green category		Number of procedures
C18	Malignant neoplasm of colon	70989
C34.8	Malignant neoplasm of overlapping sites of bronchus and lung	78623
C34.9	Malignant neoplasm of unspecified part of bronchus or lung	69795
C34	Malignant neoplasm of bronchus and lung	112450
C34.1	Malignant neoplasm: Upper lobe, bronchus or lung	41169
C50.9	Malignant neoplasm of breast of unspecified site	61159
C50	Malignant neoplasm of breast	72555
C56	Malignant neoplasm of ovary	79840
C61	Malignant neoplasm of prostate	108526
C64	Malignant neoplasm of kidney, except renal pelvis	114795
D38.1	Neoplasm of uncertain behavior of trachea, bronchus and lung	119102
D38	Neoplasm of uncertain behavior of middle ear and respiratory and intrathoracic organs	54490
G40.9	Epilepsy, unspecified	63077
G45.0	Transient Ischemic Attack	66953
G45	Transient cerebral ischemic attacks and related syndromes	57833
I63.3	Cerebral infarction due to thrombosis of cerebral arteries	121199
I63.4	Cerebral infarction due to embolism of cerebral arteries	80867
I63.5	Cerebral infarction due to unspecified occlusion or stenosis of cerebral arteries	51228
I63.8	Other cerebral infarction	43037
I63.9	Cerebral infarction, unspecified	91171
I69.3	Other paralytic syndrome following cerebral infarction	62624
J32	Chronic sinusitis	33029
K85	Acute pancreatitis	73426
R91	Abnormal findings on diagnostic imaging of lung (Solitary pulmonary nodule/ other nonspecific abnormal findings)	186000
S06.0	Concussion	53104
S06.5	Traumatic subdural hemorrhage	47408
Z51.1	Encounter for antineoplastic chemotherapy and immunotherapy	111425

Yellow category		Number of procedures
C20	Malignant neoplasm of rectum	116055
J98	Other respiratory disorders	79812
G44	Other headache syndromes	357063
G44.8	Other specified headache syndromes	162709
G54.4	Lumbosacral root disorders, not elsewhere classified	51331
G54	Nerve root and plexus disorders	202521
G98	Other disorders of nervous system not elsewhere classified	71827
I50.9	Heart failure, unspecified	55780
I69	Sequelae of cerebrovascular disease	54572

I70.2	Atherosclerosis of native arteries of the extremities	60578
M47	Spondylosis	81826
M51	Thoracic, thoracolumbar, and lumbosacral intervertebral disc disorders	88143
M54	Dorsalgia	49127
R10	Abdominal and pelvic pain	44022
R42	Dizziness and giddiness	207128
R51	Headache	193700
R55	Syncope and collapse	136701
Z03	Encounter for medical observation for suspected diseases and conditions ruled out	160208
Red category		
S00.0	Superficial head injury	257271
S00.9	Superficial injury of unspecified part of head	62502
S01.1	Open wound of scalp	96855
S00	Superficial injury of head	106255
I10	Essential (primary) hypertension	69123
